# A Novel Mobile Element ICE*Rsp*D18B in *Rheinheimera* sp. D18 Contributes to Antibiotic and Arsenic Resistance

**DOI:** 10.3389/fmicb.2020.616364

**Published:** 2020-12-18

**Authors:** Jiafang Fu, Chuanqing Zhong, Peipei Zhang, Qingxia Gao, Gongli Zong, Yingping Zhou, Guangxiang Cao

**Affiliations:** ^1^Department of Epidemiology, The First Affiliated Hospital of Shandong First Medical University, Jinan, China; ^2^College of Biomedical Sciences, Shandong First Medical University & Shandong Academy of Medical Sciences, Jinan, China; ^3^School of Municipal and Environmental Engineering, Shandong Jianzhu University, Jinan, China; ^4^Key Laboratory for Biotech-Drugs of National Health Commission, Department of Microbiology, Jinan, China

**Keywords:** antibiotic resistance, arsenic resistance, ICERspD18B, integrative and conjugative element, Rheinheimera

## Abstract

Antibiotics and organoarsenical compounds are frequently used as feed additives in many countries. However, these compounds can cause serious antibiotic and arsenic (As) pollution in the environment, and the spread of antibiotic and As resistance genes from the environment. In this report, we characterized the 28.5 kb genomic island (GI), named as ICE*Rsp*D18B, as a novel chromosomal integrative and conjugative element (ICE) in multidrug-resistant *Rheinheimera* sp. D18. Notably, ICE*Rsp*D18B contains six antibiotic resistance genes (ARGs) and an arsenic tolerance operon, as well as genes encoding conjugative transfer proteins of a type IV secretion system, relaxase, site-specific integrase, and DNA replication or partitioning proteins. The transconjugant strain 25D18-B4 was generated using *Escherichia coli* 25DN as the recipient strain. ICE*Rsp*D18B was inserted into 3'-end of the *guaA* gene in 25D18-B4. In addition, 25D18-B4 had markedly higher minimum inhibitory concentrations for arsenic compounds and antibiotics when compared to the parental *E. coli* strain. These findings demonstrated that the integrative and conjugative element ICE*Rsp*D18B could mediate both antibiotic and arsenic resistance in *Rheinheimera* sp. D18 and the transconjugant 25D18-B4.

## Introduction

In aquaculture systems, the indiscriminate use of chemical additives and antimicrobials (especially antibiotics) as preventative and curative measures for diseases has resulted in antimicrobial resistance among bacteria (Buschmann et al., 2012; Sun et al., 2016; Nakayama et al., 2017; Rico et al., 2017). Additionally, the transfer of antibiotic resistance elements from aquaculture facilities into the environment could have negative impacts on environmental biodiversity and human health as a result of further antimicrobial resistance development (Garcia-Aljaro et al., 2014; Xu et al., 2017). In addition to antibiotics, the metalloid arsenic (As) has been used as a feed additive, although it was ranked first on the priority list of hazardous substances by the Agency for Toxic Substances and Disease Registry^1^; arsenic has a significant impact on the aquaculture environment because of its toxic, persistent, and accumulative properties in organisms, which have devastating effects on the diversity of aquatic animals and on the ecological balance of aquaculture systems (Miazek et al., 2015; Rahman and De Ley, 2017). Arsenic resistance genes, usually organized in *ars* operons, have been widely identified in bacteria (Fekih et al., 2018; Serrato-Gamino et al., 2018). Therefore, the aquaculture environment poses a potential risk for the dissemination of arsenic resistance genes as well as antibiotic resistance genes (ARGs) through mobile genetic elements (Abdelhamed et al., 2019).

Bacteria of the genus *Rheinheimera* are frequently isolated from freshwater and estuaries (Baek and Jeon, 2015; Chen et al., 2019); and saline and slightly alkaline lakes (Liu et al., 2012; Zhong et al., 2014). Currently, the genus comprises 27 species.^2^ Comparative genomics analysis of *Rheinheimera* genomes revealed that the core genome is relatively small (Presta et al., 2017), which may be related to the different ecological niches colonized by members of this genus (Wang et al., 2018; Panda et al., 2020). It has been reported that many *Rheinheimera* strains are multidrug-resistant (Liu et al., 2012; Mengoni et al., 2014; Suarez et al., 2014; Kumar et al., 2015), and a series of ARGs in the genomes of *Rheinheimera* spp. have been uncovered, such as *acrD* in *Rheinheimera* sp. EpRS3, encoding an aminoglycoside efflux pump; *acrB* in *Rheinheimera* sp. KL1, encoding a multidrug resistance-nodulation-division efflux pump; and *tet*(B) in *Rheinheimera* sp. D18, encoding a tetracycline efflux major facilitator superfamily (MFS) transporter (O’Connor et al., 2015; Presta et al., 2017; Fu et al., 2020). In addition, bioinformatics analyses have predicted the widespread presence of arsenical resistance genes in *Rheinheimera*. However, the transferability of ARGs and arsenic resistance genes in *Rheinheimera* has not been well characterized.

*Rheinheimera* sp. D18 strain was previously isolated from mariculture environment in the Yellow Sea, which has been reported to be polluted by notable amounts of antibiotic residues (Du et al., 2017; Han et al., 2020) and arsenic (Jiang et al., 2015; Xiao et al., 2017), and D18 was found to have high-level resistance to tetracycline, florfenicol, amikacin, and sulfamethoxazole (Fu et al., 2020). In this study, the novel integrative and conjugative element ICE*Rsp*D18B was characterized in *Rheinheimera* sp. D18 genome. In addition to genes related to DNA replication/partitioning and conjugative transfer, ICE*Rsp*D18B was found to contain three repeated copies of a chloramphenicol/florfenicol efflux MFS transporter-encoding gene (*floR*), and several other ARGs. An arsenic tolerance operon was also identified in ICE*Rsp*D18B, indicating that ICE*Rsp*D18B mediates combined resistance to antibiotics and arsenic, and further analysis indicated that ICE*Rsp*D18B was transferable. This report characterized the first mobile genomic island (GI) ICE*Rsp*D18B that endows both antibiotic and arsenic resistance in the genus *Rheinheimera*, providing new insights into antibiotic and arsenic spread in the mariculture environment.

## Materials and Methods

### Strains and Culture Conditions

*Rheinheimera* sp. D18 strain was previously isolated from maricultural environment (Fu et al., 2020). *Rheinheimera* sp. D18 was cultured in LB solid medium (tryptone 1%, yeast extract 0.5%, 1% sodium chloride, and agar 2%) at 28°C and was used as a donor in conjugation experiments. *Escherichia coli* strain 25DN was cultured at 37°C in LB medium and was used as recipient in conjugation experiments. Transconjugants from conjugation experiments were cultured on LB medium containing florfenicol (24 mg/l) and roxarsone (8 mM) at 37°C.

### Identification of the Genomic Island

The *Rheinheimera* sp. D18 whole genome sequence has been deposited in GenBank (CP037745). The GIs were identified using Island Viewer 4 (Bertelli et al., 2017) and were further analyzed using ICEfinder (Liu et al., 2019). The genes in genomic island were annotated using the Prokaryotic Genome Annotation Pipeline on NCBI^3^ and RASTtk server (Overbeek et al., 2014; Brettin et al., 2015). Insertion sequence transposases were detected using IS-Finder (Siguier et al., 2012).

### Comparative Analysis of ICE*Rsp*D18B With Other Genetic Elements

Pairwise alignment of ICE*Rsp*D18B and other relevant genetic elements was performed using the BLAST search tool and ICEberg WU-BLAST search tool (Liu et al., 2019). Further alignment between two sequences was performed using BioXM 2.6 software.

### Conjugation Experiments

To determine whether the antibiotic and arsenic resistance genes in ICE*Rsp*D18B could be horizontal transferred among bacteria, conjugation experiments were carried out as previously described with some modification (Fu et al., 2020). Transconjugants were selected on LB agar plates with florfenicol (24 mg/l), roxarsone (8 mM), X-Gluc (5-bromo-4-chloro-3-indolyl-beta-D-glucuronic acid), and sodium azide. The donor (*Rheinheimera* sp. D18) and the recipient (*E. coli* 25DN) strains are inhibited and only the transconjugants would survive on the selective agar plates. ICE*Rsp*D18B and its insertion site in the transconjugant were demonstrated by PCR and direct DNA sequencing. The ability of ICE*Rsp*D18B to form a ring in *Rheinheimera* sp. D18 was also verified by PCR and DNA sequencing. All the primers used in this report are listed in Supplementary Table S1.

### Metalloid Arsenic and Antibiotic Susceptibility Testing

The broth microdilution method was used (CLSI, 2017) to determine the MICs for roxarsone, sodium hexafluoroarsenate and different antibiotics, including amikacin, florfenicol, and sulfamethoxazole. *Escherichia coli* 25DN strain was also tested for MICs.

### Data Analysis

All the experiments in this study were carried out in triplicate. The differences in MICs for the transconjugant strain and *E. coli* 25DN strain were analyzed using the Student’s *t*-test (*p* < 0.05).

## Results

### Structure of ICE*Rsp*D18B in the *Rheinheimera* sp. D18 Strain

**Figure 1:**
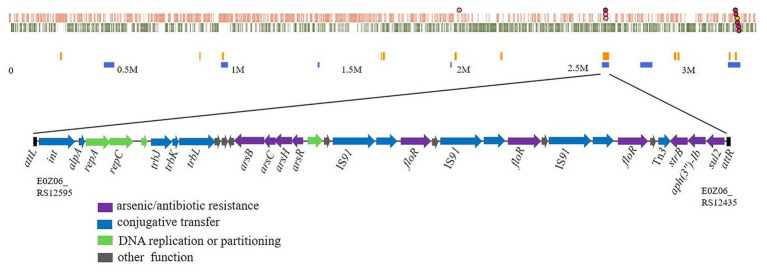
Schematic view of a new identified genomic island (GI) and its position in *Rheinheimera* sp. D18. Top image, GIs predicted by Island Viewer 4 in *Rheinheimera* sp. D18. Putative genomic islands were predicted by IslandPath-DIMOB method (blue squares) or SIGI-HMM method (orange squares). Bottom image, gene arrangement in the genomic island named ICE*Rsp*D18B. ICE*Rsp*D18B (from E0Z06_RS12595 to E0Z06_RS12435) is bordered by a 20-bp DR (5'-ACAATNGAGTGGGAATNNTT-3') in the chromosome of D18. The diagram shows the predicted classification/function of each gene (represented by arrows) as follows: violet, arsenic, or antibiotic resistance; blue, conjugative transfer; green, DNA replication or partitioning; and gray, other functions.

A chromosomal GI in *Rheinheimera* sp. D18 was identified using Island Viewer 4 (Figure 1), while it was not predicted as a typical integrative and conjugative element (ICE) by ICEfinder software. This GI extends from position 2,629,186 to 2,657,721 in the chromosome of D18 and contains 28,536 bp. Gene annotation indicated that it contains 33 open reading frames (ORFs; Supplementary Table S2), among which six ORFs were predicted to be ARGs, including one sulfonamide resistance gene (*sul2*), two aminoglycoside resistance genes (*aph(3'')-Ib* and *strB*), and three repeated copies of a chloramphenicol/florfenicol resistance gene (*floR*); and four ORFs were predicted to be arsenic resistance genes, forming the operon *arsRHCB*. The GI also contains three identical copies of a relaxase-encoding gene (E0Z06_RS12465, E0Z06_RS12485, and E0Z06_RS12505) related to a type IV secretion system; three conjugative transfer protein-encoding genes (*trbL*, *trbK*, and *trbJ*); four genes associated with DNA replication or partitioning (*repC*, *repA*, E0Z06_RS12575, and E0Z06_RS12520); and genes encoding a site-specific integrase (*int*) and its transcriptional regulator (E0Z06_RS12590). Sequence examination further indicated that the GI was bordered by a 20-bp direct repeat (DR; 5'-ACAATNGAGTGGGAATNNTT-3') at both ends and that it was inserted into the *guaA* gene (E0Z06_RS12600) in the chromosome of D18. These findings suggest that this GI might be an ICE-like genomic island, named as ICE*Rsp*D18B, and provide antibiotic and arsenic tolerance to *Rheinheimera* sp. D18, as we know, ICEs are now recognized as a large and diverse class of chromosomal mobile genetic elements in bacteria that can transfer between bacteria through conjugation (Baranowski et al., 2018; Partridge et al., 2018).

### Pairwise Alignment of ICER*sp*D18B With Relevant DNA Sequences

**Figure 2:**
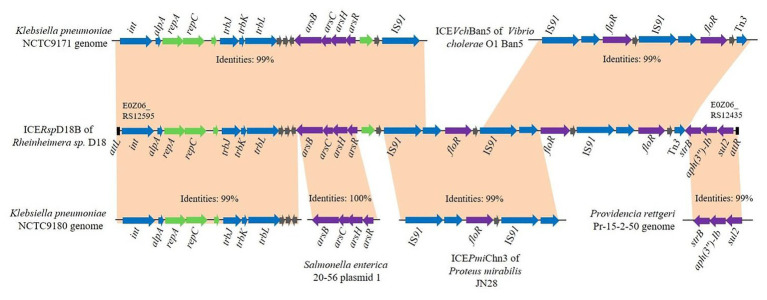
Schematic representation of the potential sources of genes in ICE*Rsp*D18B. Pairwise alignment of ICE*Rsp*D18B of *Rheinheimera* sp. D18 with closely related DNA sequences from ICE*Vch*Ban5 of *Vibrio cholerae* O1 Ban5, ICE*Pmi*Chn3 of *Proteus mirabilis* JN28, plasmid 1 of *Salmonella enterica* 20–56, and the *Klebsiella pneumoniae* NCTC9171, *K. pneumoniae* NCTC9180, and *Providencia rettgeri* Pr-15-2-50 genomes. Genes are indicated by arrows, and colors represent the following predicted functions: violet, arsenic, or antibiotic resistance; blue, conjugative transfer; green, DNA replication or partitioning; and gray, other functions. Orange shading matches regions with high sequence identity.

The whole ICE*Rsp*D18B nucleotide sequence was analyzed using BLAST, and results revealed that this ICE*Rsp*D18B presents only in the *Rheinheimera* sp. D18 genome. GC content of ICE*Rsp*D18B is 58.28%, different from that of the overall GC content of *Rheinheimera* sp. D18 genome (44.39%), indicating that this genomic island ICE*Rsp*D18B was derived from other bacteria. Pairwise alignment of ICE*Rsp*D18B with other relevant DNA sequences was performed, and the sequence alignment results are shown in Figure 2. BLASTn analysis indicated that genes relating to conjugative transfer and DNA replication or partitioning (from E0Z06_RS12595 to E0Z06_RS12545) in ICE*Rsp*D18B were highly similar to genes in the *Klebsiella pneumoniae* NCTC9180 genome (GenBank accession number LR134202.1), and these genes were also predicted to be present in the *K. pneumoniae* NCTC9171 genome (GenBank accession number LR588410.1). A larger region that included the above genes and the arsenic operon (*arsRHCB*; from E0Z06_RS12595 to E0Z06_RS12510) in ICE*Rsp*D18B showed 99% identity with a genomic region of *K. pneumoniae* NCTC9171. In addition, the ICE*Rsp*D18B arsenic operon (*arsRHCB*) had 100% nucleotide sequence identity to the arsenic operon located in *Salmonella enterica* strain 20–56 plasmid 1 (GenBank accession number LR536427.1). Of particular note, there were three tandem repeats of a set of genes that includes IS*91*, *floR*, a relaxase-encoding gene, and a LysR family transcriptional regulator-encoding gene in ICE*Rsp*D18B, one or two set of these genes were also predicted in ICE*Vch*Ban5 of *Vibrio cholerae* O1 Ban5 (GenBank accession number GQ463140) and ICE*Pmi*Chn3 of *Proteus mirabilis* JN28 (GenBank accession number KY437727). The structure of the remaining part of ICE*Rsp*D18B, including genes related to aminoglycoside and sulfonamide resistance, showed high similarity to genes in the *Providencia rettgeri* Pr-15-2-50 genome (GenBank accession number CP039844.1).

### Transfer of ICER*sp*D18B to *Escherichia coli*

**Figure 3:**
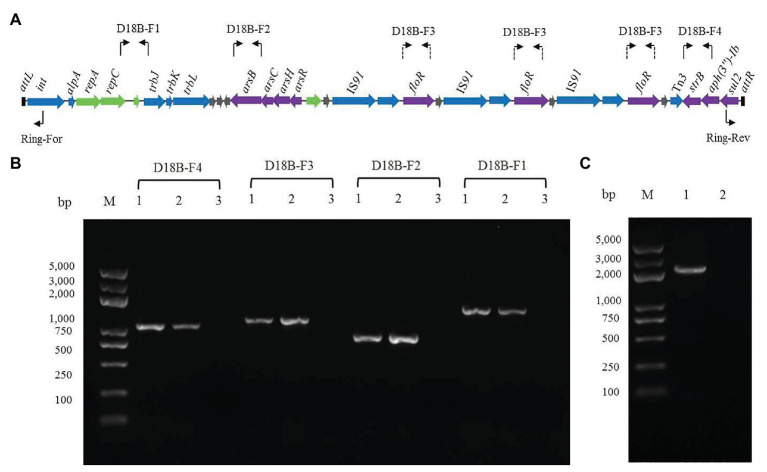
Verification of the presence and the circular form of ICE*Rsp*D18B. **(A)** Primer positions in ICE*Rsp*D18B are indicated using bent arrows. As ICE*Rsp*D18B harbors three copies of *floR*, primers for determination of the presence of *floR* are shown in three locations by dashed arrows. **(B)** Four ICE*Rsp*D18B fragments were amplified by PCR using total DNA of transconjugant 25D18-B4 (lanes 1), strain D18 (lanes 2), strain 25DN (lanes 3) as templates. **(C)** Verification of the circular form of ICE*Rsp*D18B using the primer pair Ring-For/Rev. Total DNA of strain D18 (lane 1) or strain 25DN (lane 2) was used as template. M, molecular size markers.

In order to determine whether the ICE-like chromosomal genomic island ICE*Rsp*D18B could be horizontally transferred, conjugation experiments between the donor strain D18 and the recipient strain *E. coli* 25DN (sodium azide-resistant) were performed. Florfenicol and roxarsone were used as the selective pressure, and the transconjugation frequency was about 2.76 × 10^−7^ colony-forming units/donor. One of the transconjugants was isolated and named 25D18-B4. To determine whether ICE*Rsp*D18B was inserted into the chromosome of *E. coli* 25D18-B4, PCR assays and DNA sequencing analysis were performed. The results demonstrated that genes *strB*, *floR*, and *arsB*, and the region between *repC* and *trbJ* in ICE*Rsp*D18B, were present in 25D18-B4 but not in strain 25DN (Figures 3A,B). Furthermore, these sequences had 100% identity with those of *Rheinheimera* sp. D18, revealing that ICE*Rsp*D18B had been transferred to 25D18-B4. Results also revealed that this ICE*Rsp*D18B had been excised from the chromosome and was present in a circular form in *Rheinheimera* sp. D18 (Figure 3C), which is considered to be the first step of conjugation.

### Localization of ICE*Rsp*D18B in the Transconjugant 25D18-B4

**Figure 4:**
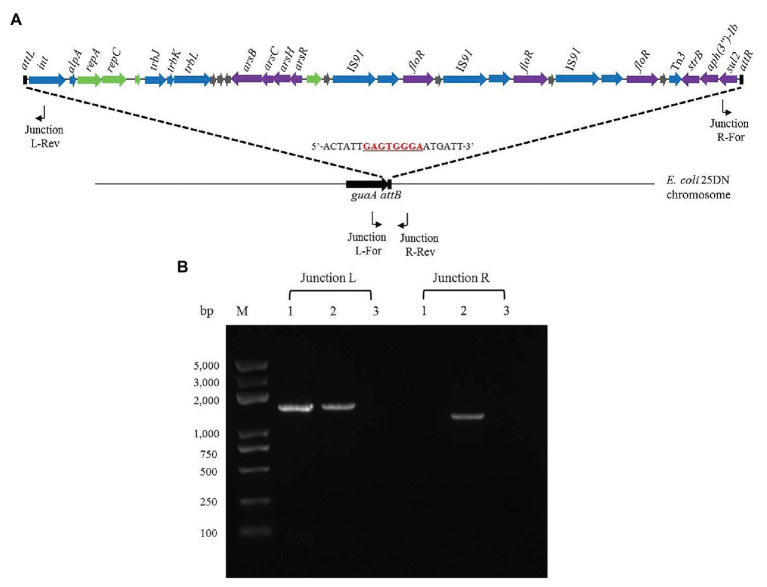
Analysis of the insertion site of ICE*Rsp*D18B in transconjugant 25D18-B4. **(A)** PCR primer positions in ICE*Rsp*D18B and in the strain 25DN chromosome are indicated by bent arrows. The insertion site of ICE*Rsp*D18B in the strain 25DN chromosome is indicated by dashed lines, and the cutting site (5'-GAGTGGGA-3') of the integrase (Song et al., 2012) is underlined and marked in red. **(B)** Gel picture of the PCR products generated by the Junction L-For/Junction L-Rev and Junction R-For/Junction R-Rev primer pairs. Total DNA of strain D18 (lanes 1), transconjugant strain 25D18-B4 (lanes 2), and strain 25DN (lanes 3) was used as template. M, molecular size markers.

The 3'-ends of tRNA/tmRNA genes are known attachment sites of ICEs (Williams, 2002; Liu and Zhu, 2010; Del Canto et al., 2011). However, the 3'-end of the guanosine monophosphate synthetase-encoding gene *guaA* has also been reported as an insertion site of genomic islands (Song et al., 2012). As bioinformatics analysis had indicated that ICE*Rsp*D18B was inserted into 3'-end of *guaA* in the *Rheinheimera* sp. D18 genome, we investigated its location in the transconjugant 25D18-B4 and whether integration was orientation-specific, using PCR and DNA sequencing. 25D18-B4 was analyzed by PCR using combinations of two primer pairs: Junction L-For/Junction L-Rev and Junction R-For/Junction R-Rev, with D18 and *E. coli* 25DN as controls (Figure 4). It should be noted that the sequence of the Junction L-For primer is also present in the *guaA* gene of D18, due to the high similarity of *guaA* in D18 and 25DN, and that Junction L fragments were amplified in both 25D18-B4 and D18 (Figure 4B). PCR results indicated that ICE*Rsp*D18B had been inserted into the 3'-end of *guaA* gene of the transconjugant 25D18-B4 strain, and DNA sequence analysis of PCR products confirmed that ICE*Rsp*D18B was inserted at this site.

### Susceptibility of D18 and 25D18-B4 to Antibiotics and Arsenic

The susceptibility of transconjugant 25D18-B4 and *Rheinheimera* sp. D18 to metalloid arsenic and antibiotics was tested. As shown in Table 1, 25D18-B4 had acquired resistance to florfenicol (MIC, 92 mg/L), amikacin (MIC, 24 mg/L), sulfamethoxazole (MIC, 16 mg/L), sodium hexafluoroarsenate (MIC, 22 mM), and roxarsone (MIC, 14 mM). MIC testing revealed that the MICs for amikacin, florfenicol, sulfamethoxazole, sodium hexafluoroarsenate, and roxarsone in the transconjugant 25D18-B4 were higher than the MICs for the recipient strain 25DN (Table 1). The notable increase in antibiotic/arsenic resistance of 25D18-B4 suggested that ICE*Rsp*D18B genes involved in antibiotic and arsenic resistance had been horizontally transferred to the *E. coli* strain.

**Table 1:** MICs of antibiotics and arsenic (As).

Strain	Amikacin^*^	Florfenicol	Sulfamethoxazole	Roxarsone^#^	Sodium hexafluoroarsenate^#^
D18	96	128	72	20	35
25DN	<2	<2	4	4	10
25D18-B4	24	92	16	14	22

* Concentrations of the three antibiotics are given in mg/L.

# Concentrations of roxarsone and sodium hexafluoroarsenate are given in mM.

## Discussion

In this study, we reported the discovery and characterization of the ICE-like chromosomal genomic island ICE*Rsp*D18B in the genus *Rheinheimera*. BLASTn analysis indicated that only part sequence of ICER*sp*D18B exists in other species, and mainly derived from pathogenic bacteria such as *Vibrio cholerae*, *K. pneumoniae*, and *P. rettgeri* (Figure 2). Further alignment with ICEberg WU-BLAST search tool revealed that the overall nucleotide sequence of ICE*Rsp*D18B has low similarity to that of previously described ICEs, although a portion of ICE*Rsp*D18B showed high similarity to ICE*Vch*Ban5 of *Vibrio cholerae* O1 Ban5 and ICE*Pmi*Chn3 of *P. mirabilis* JN28 (Figure 2). Additionally, our conjugation experiments indicated that ICE*Rsp*D18B has the ability to transfer among bacteria. Hence, we speculate that ICE*Rsp*D18B was transferred horizontally from other unsequenced strains. Moreover, ICE*Rsp*D18B contains genes predicted to encode a site-specific integrase, relaxases associated with a type IV secretory pathway, conjugative transfer proteins, and DNA replication or partitioning encoding genes (Supplementary Table S2), further suggesting that ICE*Rsp*D18B is an ICE.

tRNA, tmRNA, and some small RNA genes are recognized as integration hotspots of genomic islands (Williams, 2002; Del Canto et al., 2011). However, the 3'-end of the *guaA* gene is also an insertion site of genomic islands (Song et al., 2012). Integrases in *guaA*-associated genomic islands are frequently phage P4 integrases, and genes encoding AlpA (the positive regulatory protein of P4 integrases) are located near the P4 integrase genes in these genomic islands (Song et al., 2012). The 8-bp consensus sequence 5'-GAGTGGGA-3' within the DR flanking these genomic islands was reported to be the cutting site of the P4 integrases (Song et al., 2012). In our study, bioinformatics analysis revealed that the site-specific integrase in ICE*Rsp*D18B belongs to the phage P4 integrases and that the AlpA-encoding gene *alpA* is next to the site-specific integrase-encoding gene *int* (Supplementary Table S1). Additionally, the 8-bp consensus sequence 5'-GAGTGGGA-3' was also found within the DR (5'-ACAATNGAGTGGGAATNNTT-3') of ICE*Rsp*D18B, and ICE*Rsp*D18B was confirmed to be inserted into the 3' end of *guaA* in the transconjugant 25D18-B4 (Figure 4). In addition, the circular, extrachromosomal form of ICE*Rsp*D18B was also observed in *Rheinheimera* sp. D18 using PCR (Figure 3). These data suggest that ICE*Rsp*D18B was first excised from the donor *Rheinheimera* sp. D18 chromosome, transferred *via* type IV secretory system-mediated conjugation and then inserted into 3'-end of *guaA* gene of the *E. coli* 25DN chromosome by site-specific recombination. These data also indicated that ICE*Rsp*D18B has the ability to transfer genes horizontally from *Rheinheimera* sp. D18 to other bacteria. Considering that ICE*Rsp*D18B is also located at the 3'-end of *guaA* in the *Rheinheimera* sp. D18 genome, our results further demonstrate that the 3'-end of *guaA* gene may be another integration hotspot of genomic islands.

Organoarsenic arsenical compounds (such as p-arsanilic acid and roxarsone) are widely used as feed additives in many countries, and the land application of poultry or swine litter could cause serious arsenic pollution in the environment (Liang et al., 2014; Xie and Cheng, 2019), potentially resulting in arsenic resistance among environmental bacteria and the dissemination of their arsenic resistance genes to other bacterial species. Arsenic resistance genes are usually organized in *ars* operons in bacteria, such as in *Pseudomonas putida*, which has two *arsRBCH* operons and which is highly resistant to organoarsenicals and inorganic arsenic (Canovas et al., 2003; Villadangos et al., 2012). The *arsB* gene encodes an As(III) efflux permease, a*rsC* encodes an arsenate reductase for reduction of inorganic arsenate to As(III) and *arsR* encodes an As(III)-responsive transcriptional factor that controls expression of the operon (Yang et al., 2012). Arsenate [As(V)] is reduced to arsenite [As(III)] by the arsenate reductase ArsC prior to efflux, and then, arsenite is pumped out through ArsB (Shen et al., 2013). *arsH* encodes an organoarsenical oxidase that confers resistance to organoarsenic (Chen et al., 2015; Xie and Cheng, 2019). ICE*Rsp*D18B contains one *ars* gene cluster, which includes *arsBCHR* (Figure 1). The transconjugant 25D18-B4, which acquired ICE*Rsp*D18B, was found to have markedly higher MICs of roxarsone and sodium hexafluoroarsenate compared to those of the parental strain, *E. coli* 25DN (Table 1). These data suggest that ICE*Rsp*D18B can contribute to the dissemination of arsenic resistance genes among bacteria.

Sulfonamide, chloramphenicol/florfenicol, and aminoglycoside have been used widely to treat bacterial and protozoan infections in aquaculture systems (Dang et al., 2007; Hoa et al., 2008). ICE*Rsp*D18B also contains three copies of a chloramphenicol/florfenicol efflux MFS transporter-encoding gene (*floR*); one sulfonamide resistance gene (*sul2*); and two aminoglycoside resistance genes, *aph(3'')-Ib*, and *strB*. *Escherichia coli* is an opportunistic bacterium that can cause a wide variety of intestinal and extraintestinal infections (Riley, 2014). In this study, ICE*Rsp*D18B was horizontal transferred to *E. coli* 25DN strain, and generated the transconjugant 25D18-B4 strain. The transconjugant 25D18-B4 was found to have notably higher MICs of amikacin, florfenicol, and sulfamethoxazole when compared to the parental strain, *E. coli* 25DN (Table 1), suggesting that the ARGs in ICE*Rsp*D18B contribute to the antibiotic resistance profile of *Rheinheimera* sp. D18 as well as of *E. coli* 25D18-B4. These data suggest that the ICE-like genomic island ICE*Rsp*D18B has the ability to disseminate these ARGs, along with arsenic resistance genes, among bacteria in the environment.

In conclusion, the findings of this study demonstrate that ICE*Rsp*D18B is an ICE that increases host tolerance to arsenic and several antibiotics. Our results also reveal that this mobilizable ICE*Rsp*D18B could be horizontal transferred to *E. coli* 25DN strain, and the transconjugant 25D18-B4 also has resistance to arsenic and antibiotic. Continuous monitoring of the antibiotic/arsenic tolerance of bacteria detected in the aquaculture industry is recommended to reduce the spread of resistance genes.

## Data Availability Statement

The original contributions presented in the study are included in the article/Supplementary Material, further inquiries can be directed to the corresponding author.

## Author Contributions

JF: executed the experiments and manuscript preparation and submission. CZ: resources, review and editing. PZ and GZ: data curation and investigation. YZ and QG: methodology. GC: designed the work and revised the manuscript. All authors contributed to the article and approved the submitted version.

### Conflict of Interest

The authors declare that the research was conducted in the absence of any commercial or financial relationships that could be construed as a potential conflict of interest.

## Footnotes

^1^https://www.atsdr.cdc.gov/spl/index.html

^2^http://www.bacterio.net/rheinheimera.html

^3^http://www.ncbi.nlm.nih.gov/genome/annotation_prok/
