# Supplementary material for: A Novel Mobile Element ICERspD18B in Rheinheimera sp. D18 Contributes to Antibiotic and Arsenic Resistance
Source: Front Microbiol. 2020 Dec 18;11:616364. doi: 10.3389/fmicb.2020.616364 (PMC7775301; doi:10.3389/fmicb.2020.616364)
Supplement: Supplementary file 1 [file Data_Sheet_1.docx]

**Supplementary Material**

**A novel mobile element ICE*Rsp*D18B** **in *Rheinheimera* sp. D18 contributes to antibiotic and arsenic resistance**

Jiafang Fu ^a,b^, Chuanqing Zhong ^c^, Peipei Zhang ^a,b,d^, Qingxia Gao ^b^, Gongli Zong ^a,b,d^, Yingping Zhou ^c^, Guangxiang Cao ^a,b,d,*^

^a^ Department of Epidemiology, the First Affiliated Hospital of Shandong First Medical University, Jinan 250117, China

^b^ College of Biomedical Sciences, Shandong First Medical University & Shandong Academy of Medical Sciences, Jinan 250117, China

^c^ School of Municipal and Environmental Engineering, Shandong Jianzhu University, Jinan 250101, China

^d^ Key Laboratory for Biotech-Drugs of National Health Commission, Jinan 250117, China

* Corresponding author: Guangxiang Cao, Department of Epidemiology, the First Affiliated Hospital of Shandong First Medical University, Shandong Medicinal Biotechnology Center, Shandong First Medical University & Shandong Academy of Medical Sciences, Qingdao Road 6699, Jinan 250117, Shandong, P.R. China. Tel.: +86 531 8291 9606; Fax: +86 531 8291 9645

**Running title:**

ICE*Rsp*D18 mediate antibiotic/arsenic resistance

**Table S1** Primers used in this study.

| **Oligonucleotide** | **DNA Sequence (5’→3)** |
| --- | --- |
| D18B-F1-For | CGGTGGTAGTGGTGATAAAG |
| D18B-F1-Rev | AGTGAACGAATACGCCAAGG |
| D18B-F2-For | AAGACCGAGCAGGAAGCATG |
| D18B-F2-Rev | AAGGACAGGCGACAGAGTGC |
| D18B-F3-For | AGCCCCAACGAAACCAGTAC |
| D18B-F3-Rev | TCGCCCGGTATTCCTTAATC |
| D18B-F4-For | GACCCTAAAACTCTTCAATG |
| D18B-F4-Rev | AGGAGAAGTCGCTTGATATC |
| Ring-For | CGTTTCATCCGCTCCTTTGG |
| Ring-Rev | AGCAAATCCGCTCCAGACAG |
| Junction L-For | CACCACAACGTGGGTGGC |
| Junction L-Rev | CTCTTGCGCACCATCGTC |
| Junction R-For | CGAAACAGACAGAAGCACCG |
| Junction R-Rev | AGAAGAAGCCCGCGTTAAGC |

**Table S2** Genes annotated in ICE*Rsp*D18B of *Rheinheimera* sp. D18.

| Name | Start | End | Strand | gene | Function |
| --- | --- | --- | --- | --- | --- |
| E0Z06_RS12435 | 2629495 | 2630310 | + | *sul2* | sulfonamide-resistant dihydropteroate synthase Sul2 |
| E0Z06_RS12440 | 2630371 | 2631174 | + | *aph(3'')-Ib* | aminoglycoside O-phosphotransferase APH(3\'\')-Ib |
| E0Z06_RS12445 | 2631174 | 2632010 | + | *strB* | aminoglycoside O-phosphotransferase APH(6)-Id |
| E0Z06_RS12450 | 2631982 | 2632521 | - | Tn*3* | IS91 family transposase |
| E0Z06_RS12455 | 2632633 | 2632938 | - |  | LysR family transcriptional regulator |
| E0Z06_RS12460 | 2633022 | 2634236 | - | *floR* | chloramphenicol/florfenicol efflux MFS transporter FloR |
| E0Z06_RS12465 | 2634453 | 2635337 | - |  | Type IV secretory pathway, relaxase |
| E0Z06_RS12470 | 2635368 | 2636861 | - | IS*91* | IS91 family transposase |
| E0Z06_RS12475 | 2636973 | 2637278 | - | - | LysR family transcriptional regulator |
| E0Z06_RS12480 | 2637248 | 2638519 | - | *floR* | chloramphenicol/florfenicol efflux MFS transporter FloR |
| E0Z06_RS12485 | 2638736 | 2639620 | - |  | Type IV secretory pathway, relaxase |
| E0Z06_RS12490 | 2639651 | 2641142 | - | IS*91* | IS91 family transposase |
| E0Z06_RS12495 | 2641208 | 2641560 | - |  | LysR family transcriptional regulator |
| E0Z06_RS12500 | 2641588 | 2642799 | - | *floR* | chloramphenicol/florfenicol efflux MFS transporter FloR |
| E0Z06_RS12505 | 2643016 | 2643837 | - |  | Type IV secretory pathway, relaxase |
| E0Z06_RS12510 | 2643931 | 2645424 | - | IS*91* | IS91 family transposase |
| E0Z06_RS12515 | 2645635 | 2645859 | - |  | hypothetical protein |
| E0Z06_RS12520 | 2645856 | 2646593 | - |  | resolvase |
| E0Z06_RS12525 | 2646741 | 2647073 | + | *arsR* | helix-turn-helix transcriptional regulator |
| E0Z06_RS12530 | 2647070 | 2647837 | + | *arsH* | arsenical resistance protein ArsH |
| E0Z06_RS12535 | 2647834 | 2648340 | + | *arsC* | arsenate reductase ArsC |
| E0Z06_RS12540 | 2648337 | 2649404 | + | *arsB* | arsenite efflux transporter |
| E0Z06_RS12545 | 2649519 | 2649722 | + |  | hypothetical protein |
| E0Z06_RS12550 | 2649759 | 2649983 | - |  | hypothetical protein |
| E0Z06_RS12555 | 2650047 | 2650295 | - |  | hypothetical protein |
| E0Z06_RS12560 | 2650300 | 2651727 | - | *trbL* | P-type conjugative transfer protein TrbL |
| E0Z06_RS12565 | 2651738 | 2651974 | - | *trbK* | entry exclusion lipoprotein TrbK |
| E0Z06_RS12570 | 2651987 | 2652766 | - | *trbJ* | P-type conjugative transfer protein TrbJ |
| E0Z06_RS12575 | 2652907 | 2653095 | - |  | stabilization protein |
| E0Z06_RS12580 | 2654128 | 2655009 | - | *repC* | replication protein C |
| E0Z06_RS12585 | 2654996 | 2655823 | - | *repA* | replication protein A |
| E0Z06_RS12590 | 2655828 | 2656049 | - | *alpA* | phage regulatory protein AlpA |
| E0Z06_RS12595 | 2656197 | 2657399 | - | *int* | site-specific integrase |
| **E0Z06_RS12600*** | **2657704** | **2659281** | **-** | ***guaA*** | **glutamine-hydrolyzing GMP synthase** |

* ICE*Rsp*D18B contains genes from E0Z06_RS12435-E0Z06_RS12595, and was inserted into 3’-end of *guaA* gene (E0Z06_RS12600).
